# Bioresorbable electrodes in implantable electronic healthcare devices

**DOI:** 10.3389/fbioe.2025.1666446

**Published:** 2025-11-26

**Authors:** Bogdana Đorđević, Larysa Baraban, Željko Janićijević

**Affiliations:** 1 Institute of Radiopharmaceutical Cancer Research, Helmholtz-Zentrum Dresden-Rossendorf e. V. (HZDR), Dresden, Germany; 2 Else Kröner-Fresenius Center for Digital Health (EKFZ), Technische Universität Dresden (TU Dresden), Dresden, Germany

**Keywords:** degradable electrodes, electrode-tissue interfacing, bioresorbable conductive composites, triggered degradation, electroceuticals, healthcare monitoring, implantable electronic devices, transient bioelectronics

## Abstract

Implantable electronic devices for local *in vivo* monitoring of health parameters are an invaluable supplement to traditional diagnostic tools, offering real-time and personalized assessment. Such devices can be used to deliver different formats of patient-tailored treatment via controlled drug delivery or targeted stimulation. While non-degradable electronic healthcare devices are often challenging to interface with soft tissues and introduce risks associated with surgical extraction procedures, bioresorbable electronics–electronics that is safely decomposed and physiologically absorbed in the human body–undergoes controlled degradation and thus offers a promising approach for temporary monitoring and therapy. The crucial components of temporary bioelectronics are bioresorbable electrodes–electrically conductive interfaces that can be safely resorbed in the body–that should provide seamless integration with surrounding tissues, reliable functionality, and sufficient electromechanical integrity during their operational lifetime. Such electrodes find use as physical and chemical sensing elements, stimulator interfaces, and drug delivery modulators. Advances in materials science have led to significant milestones, such as 1) highly localized electrode-tissue interfacing, 2) monitoring of moving organs and less invasive implantation, and 3) electrochemical sensing with interference and degradation compensation. Additionally, integrated bioresorbable power sources and photo- or acoustically induced modulation have obviated the need for physical interconnects with external components. This mini review provides core insights into the emerging applications of bioresorbable electrodes for sensors, electroceuticals, and multifunctional devices combining sensing with electrotherapy, optogenetic stimulation, and/or drug delivery. We focus on application-specific materials selection and discuss the perspectives for improving the design and development of bioresorbable electronic healthcare devices.

## 1 Introduction

Implantable electronic devices are important clinical tools, commonly used to perform invasive electrophysiological recordings and electrical stimulation of tissues within the nervous, cardiovascular, and musculoskeletal systems. The significance of these devices as targeted electroceuticals and healthcare monitoring systems is continuously increasing with the popularization of point-of-care concepts involving (bio)chemical sensing and theranostics. Traditional implantable electronics is permanent, rigid, and bioinert, limiting seamless integration with biological tissues and increasing risks for the patient due to long-term implantation effects and surgical device retrieval. In many clinical scenarios, implantable electronics should fulfill a temporary monitoring and/or therapeutic function, favoring soft, flexible, and transient (i.e., degradable) devices that decompose in the body. For safe use, implanted electronic devices must be bioresorbable (harmlessly physiologically absorbed by the body). Bioresorbable implantable electronic devices can improve tissue interfacing and unlock the opportunity to effectively address temporary post-surgical monitoring or diagnostic applications (Biesmans et al., 2024; Yang et al., 2022; Zhang et al., 2025) and incorporate spatiotemporally controlled therapy, e.g., via electrical stimulation and programmable drug delivery (Wang et al., 2025; Zhang et al., 2023a). These devices reduce the implantation risks for the patient, decrease surgical complexity, and lower healthcare costs by obviating the need for surgical device removal. In addition, their physiological biodegradation helps to minimize the amount of electronic waste generated in healthcare, thereby supporting sustainability and circular economy principles.

The key interfacing components in bioresorbable implantable electronics are bioresorbable electrodes, designed to achieve desired transiency, integration with living tissues, reliable functionality, and electromechanical integrity. There are multiple reviews covering broadly the materials and construction of bioresorbable electronic devices (Hwangbo et al., 2024; Sacchi et al., 2024; Janićijević et al., 2024; Lee, et al., 2023), as well as more specific aspects of transient power supplies (Kang et al., 2023), sensors (Hu et al., 2024), and bioelectronic neural interfaces (Wang et al., 2024). Although they discuss bioresorbable electrodes in specific contexts, a systematic assessment of key features falls out of their scope. Our mini review focuses on the materials, design, and performance aspects of bioresorbable electrodes in diverse applications to concisely provide core insights to researchers developing electrodes for bioresorbable electronic systems. We provide a brief overview of key publications with notable innovative contributions to the field of bioresorbable electrodes for healthcare devices in Table 1.

**TABLE 1:** Key advances in bioresorbable electrodes for implantable healthcare devices.

Reference	Materials	Geometry	Target use	Control interface and power supply	Operational lifetime	Features
Wei et al. (2022)	Candelilla wax, Mo or W microparticles	Planar structure (2D) with conductive traces patterned into the substrate	Temperature sensors, capacitive proximity sensors, interconnects, filters	External control and power supply	19 days until electrical failure (in PBS^a^ at 37 °C)	+ Facile fabrication + Non-toxic solvents - Brittle structure - Difficult to interface with external electronics
Kim et al. (2022)	PBAT^b^, tetraglycol, and Mo microparticles	Conductive paste for printing of electrodes with arbitrary geometry	Strain sensors, resistive heaters, inductive coils, interconnects	External control and power supply	16 days until complete degradation (in PBS at 37 °C)	+ High stretchability + Compliance with various electrode patterning approaches
Fumeaux and Briand (2024)	POMaC^c^ and shellac-carbon ink	Planar, 3D printed electrodes as well as soft, structured surfaces	Pressure sensors, strain sensors, electrode arrays for electrophysiological monitoring	External control and power supply	Several months until complete degradation (in PBS at 37 °C)	+ Customizable additive manufacturing based on medical imaging + Comparable electrical performance with commercial electrodes - Toxic solvents
Shin et al. (2024)	PGCL^d^, PEDOT:PSS^e^, and D-sorbitol	Planar or fiber electrodes	Temperature sensor, wireless, thermally actuated drug delivery	External control and power supply	12 weeks until complete degradation (in PBS at 37 °C)	+ Highly stretchable + Suitable as a substrate, encapsulation or, if doped, as a conductor
Jang et al. (2024)	PEDOT:PSS doped with PEG^f^, glycerol, and 1-butyl-1-methylpyrrolidinium bis(trifluoromethylsulfony) imide (P14 [TFSI]) (PLCL-IPDI-AFD)	Patterned, planar electrode arrays with self-healing properties	Humidity and temperature sensors, tactile sensors, monitoring and manipulation of moving organs (e.g., urinary bladder)	External control and power supply	28 days until complete degradation (in PBS at 37 °C)	+ Fast autonomous restoration of electrical function after breaks
Kim et al. (2023a)	PCL^g^ with embedded Mo microparticles	Fiber (1D)	Interconnects, deep wound temperature sensor, nerve and muscle stimulator	External control and power supply	77 days for 42% mass decrease (in PBS at 37 °C)	+ Facile fabrication - Possible fragmentation-induced complications
Prieto et al. (2024)	PCL/PLGA^h^ and Mo	Fiber (1D)	Temporary epicardial pacing wire	External control and power supply	Only 5% of mass of uncoated leads lost after 28 days (tested in simulated body fluid)	+ Compatible with commercial pacing hardware - Possible fragmentation-induced complications
Istif et al. (2024)	cPPA^i^ and resorbable metals (e.g., Mo, Fe, Zn, Mg)	Planar (2D) metal electrodes supported and encapsulated by cPPA	Capacitive and resistive sensors	External control and power supply	Wireless photo-triggered degradation, with subsequent dissolution in alkaline PBS solution (pH 14)	+ External modulation of degradation - Possible fragmentation-induced complications
Lee et al. (2023)	PHBV^j^, PEG, and ChCl^k^	Planar structure rolled into a cylinder	Peripheral nerve stimulator	Controlled and powered through externally applied acoustic waves	Stable for 5 days *in vivo* before a wireless acoustically triggered degradation with subsequent dissolution	+ External modulation of degradation and operation + Compatible with commercial acoustic sources
Strakosas et al. (2023)	ETE^l^ with 2-ethoxyacetic acid sodium salt side chain, PVA^m^, PLL^n^, horseradish peroxidase (HRP^o^), oxidase enzymes, and EDC/sulfo-NHS^p^	3D substrate-free electrode, self-assembled *in vivo*	Intimate interfaces with neural tissue for highly localized electrotherapy	External control and power supply	Systematic testing not conducted	+ Seamless interfacing with soft neural tissues - Degradation behavior *in vivo* not investigated - Difficult to interface with external electronics
Rosenbalm et al. (2023)	PGSA^q^ and PPy^r^	Planar structured surface rolled into a cylinder	Peripheral nerve stimulator	External control and power supply	Up to a year *in vivo* until degradation	+ Passive or active stimulation possible + Facile fabrication - Difficult to interface with external electronics
Yu et al. (2024)	PLLA^s^-PTMC^t^, PCL, Zn, Mo	Multilayered cylinder with aligned polymer fibers for nerve growth guidance	Peripheral nerve stimulator	Integrated bioresorbable galvanic cell electrodes for continuous nerve stimulation	33 days until complete dissolution (tested in PBS at 37 °C for 7 days, and then at 65 °C)	+ Self-powered operation + Mechanical guidance of nerve growth - Lack of controlled stimulation
Kim et al. (2023b)	PCL and Mo microparticles	Double-layered cylinder	Peripheral nerve stimulator	Wireless external control and power supply	Conductivity preserved up to 30 days (in PBS at 37 °C)	+ Composite material also applicable for electrode printing and tissue engineering - Non-degradable realization of inductive coupling and power supply
Zhang et al. (2025)	Polyanhydride, doped Si, SiO_2_, Candelilla wax, W, Mg, Zn, MoO_3_	Millimeter-sized, grain-like, rectangular structure	Cardiac pacing or multisite, programmable tissue stimulation	Controlled through externally applied light, self-powered through integrated galvanic cell electrodes	Functionality retained *in vivo* for up to 6 days (20 days) for Mg–MoO_3_ (Zn–MoO_3_)	+ Miniature, facilitated integration with surgical procedures + Modular assembly for multisite stimulation -Possible obstructions due to degradation products or device migration
Lee et al. (2024)	PCL, Mo, MoO_3_, PBAT-wax	Planar (2D) electrodes for piezoresistive measurements facilitated by surface cracks	Small-strain monitoring in tissues (cardiac pulsatility, muscular contraction/relaxation, hemodynamic state during surgery)	External control and power supply	Stable operation up to 3 days *in vivo*	+ Highly sensitive to small strains in tissues - Surface cracks accelerate degradation
Li et al. (2024)	PDPAEMA-PEGDA^u^, Candelilla wax, beeswax, PLGA, PVA, Zn, W	Hydrogel-embedded serpentine metal inductor connected to a wax-encapsulated planar capacitor	Gastric leakage monitoring	Sensing information collected through inductive coupling, power supply not needed	Stable operation up to 7 days *in vivo*	+ Fast response to stomach acid leaks (up to 1 h) - Low sensitivity, nonlinear response, slow degradation
Bae et al. (2024)	PLCL^v^-PLGA, SiO_2_, Si, Mg, Mo	Web-like polymer support with electrode array positions at nodes and ends	Brain cortex interfacing	Wireless external control and power supply of the implanted NFC (near-field communication) module	Completely degraded after 460 days *in vivo*	+ Facilitated self-deployment through a syringe in the intracranial space - Possible crippling failure during insertion - Non-degradable NFC module
Yang et al. (2022)	PLGA, Si, SiO_2_, MoS_2_/WS_2_, Fe nanoparticles	Planar, electrode arrays patterned on a polymer substrate	Temperature sensor, pH sensor, electrophysiological monitoring, dopamine sensor	External, wirelessly powered wearable control module	Stable operation for ≈2–4 weeks, followed by gradual degradation *in vivo*	+ Highly localized investigation of deep brain regions - Expensive fabrication process
Li et al. (2023a)	PLGA, Mo, Zn	Planar, electrode arrays patterned on a polymer substrate	Glucose sensor	External, battery-powered wearable control module	5 days of operation; 2 months until near complete dissolution (in PBS at room temperature and *in vivo*)	+ Measurements compensated for degradation with comparable accuracy to commercial implantable glucose monitors - Possible complications due to the accumulation of degradation products during chronic use
Wang et al. (2025)	PLLA-PTMC, Si, Mg, FeMn, Mo	Cylindrical nerve conduit	Peripheral nerve stimulator	Integrated bioresorbable galvanic cell electrodes for continuous nerve stimulation and an electrode array for nerve growth mapping	5 weeks of stable operation *in vivo*	+ Simultaneous support and precise real-time monitoring of nerve growth and early detection of neuromas - Lack of controlled stimulation - Interfacing with external electronics through non-degradable wires
Cho et al. (2024)	PLGA, Si, SiO_2_, Mo	Mesh microelectrodes integrated with waveguides on a planar support	Hybrid neural implant for electrophysiological recording and optogenetic stimulation	External control and power supply	3 weeks of stable operation *in vivo*	+ Colocalized monitoring and stimulation of the cerebral cortex - Complex interfacing with external electronics and light sources
Chen et al. (2023)	PLGA, Mo	Transparent mesh microelectrode array on a planar substrate	Multisite pacing and spatiotemporal cardiac function mapping with a possibility of coupling with optical mapping	External control and power supply	Less than 2 weeks *in vivo* until failure	+ Uniform electrical performance comparable with commercial solutions - Expensive and long fabrication process
Huang et al. (2022)	PLGA, Mg, W	Polymer microneedles coated with a thin metal layer	Passive drug delivery combined with electrotherapy for muscle regeneration	External wireless control and power supply through inductive coupling	Several days of stable operation *in vivo*; several months until complete bioresorption	+ Synergistic effect of active stimulation and pharmaceuticals accelerates healing - Relatively short functional lifespan
Zhang et al. (2023a)	Polyanhydride, Candelilla Wax/W, (Mg, Mg-SOG^w^ or Zn), (Fe, Mo, W or MoO_3_)	Polymer reservoirs with planar electrodes as release valves	Externally programmed drug delivery	External photo-triggered reservoir opening through the corrosion of galvanic cell electrodes	Not extensively investigated	+ Addressable reservoirs and externally programmable drug delivery - Limited spatial resolution of drug reservoir activation
Kaveti et al. (2024)	PLCL, ZnO/PLCL, Mg	Planar multilayer structure reinforced with polymer mesh	Hernia repair support with drug delivery and wireless pressure monitor	Wireless post-operative monitoring and triggered drug delivery	6 weeks for electrical components to degrade and up to 25 weeks of preservation of mechanical support (in PBS at 37 °C)	+ Synergy of mechanical support with drug delivery reduces the risk of hernia recurrence

^*a*^ PBS, phosphate-buffered saline.

^*b*^ PBAT, polybutylene adipate terephthalate.

^*c*^ POMaC - poly (octamethylene maleate (anhydride) citrate).

^*d*^ PGCL, poly (caprolactone-co-glycolide).

^*e*^ PEDOT:PSS, poly (3,4-ethylenedioxythiophene) polystyrene sulfonate.

^*f*^ PEG, polyethylene glycol.

^*g*^ PCL, poly-ε-caprolactone.

^*h*^ PLGA, poly (lactic-co-glycolic) acid.

^*i*^ cPPA, cyclic poly (phthalaldehyde).

^*j*^ PHBV, poly (3-hydroxybutyrate-co-3-hydroxyvalerate).

^*k*^ ChCl - choline chloride.

^*l*^ ETE, 2,5-bis(2,3-dihydrothieno[3,4-b][1,4]dioxin-5-yl)thiophene.

^*m*^ PVA, poly (vinylalcohol).

^*n*^ PLL, poly-L-lysine.

^*o*^ HRP, horseradish peroxidase.

^*p*^ EDC/sulfo-NHS, 1-ethyl-3-[3-dimethylaminopropyl]carbodiimide/N-hydroxysulfosuccinimide.

^*q*^ PGSA, poly (glycerol sebacate) acrylate.

^*r*^ PPy, poly (pyrrole).

^*s*^ PLLA, poly (L-lactic acid).

^*t*^ PTMC, poly (trimethylene carbonate).

^*u*^ PDPAEMA-PEGDA, poly [2-(diisopropylamino)ethyl methacrylate]-poly (ethylene glycol) diacrylate.

^*v*^ PLCL, poly (lactide-co-ε-caprolactone).

^*w*^ SOG, spin-on-glass.

## 2 Bioresorbable composites for bioelectronics

Development of transient bioelectronic implants relies on characterizing the effect of electrode degradation products on tissue health. Passive degradation pathways of some metals were previously researched for the use in stents and orthopedic implants. Iron slowly degrades in physiological environments, forming hydroxides and oxides that could induce formation of reactive oxygen radicals, promoting tissue injury (Li Jiameng et al., 2023). Fast magnesium dissolution yields harmless oxides and phosphates but also the potential buildup of hydrogen gas (Singh et al., 2024). Tungsten is widely used in medical implants; however, it converts to soluble oxide species that are reported to be toxic to cells (Idil and Donaldson, 2018). In terms of biocompatibility, promising metals for bioresorbable electrodes are zinc and molybdenum, both producing semipassivating oxide layers which dissolve into fully resorbable ions–Zn^2+^ and molybdate–within weeks or months (Yang et al., 2017; Redlich et al., 2020). *In vivo* toxicity (LD_50_) values obtained via oral supplementation for iron, magnesium and zinc are 1,300, 5,000 and 350 mg/kg, respectively, classifying them as low-toxic. Amounts of trace elements Mo and W in thin-film electrodes are more strictly limited as the recommended daily intakes are in the order of 10 μg (Ryu et al., 2021). However, acute toxicity is not a sufficient indicator for evaluating the risks associated with prolonged, localized exposure due to dissolution. Reduced metal content and tuning of degradation rates can alleviate chronic toxicity effects. To modulate corrosion, accumulation of ions, and mechanical fragmentation, different encapsulation and composite formulations with polymers are being developed. Solvent casting and additive manufacturing are used for producing soft, thin, and flexible films, showing improved tissue conformity (Janićijević et al., 2024). Other techniques (e.g., electrospinning) are used to create structured surfaces, providing mechanical support for tissue growth (Yu et al., 2024). In metal-polymer systems, conductivity is achieved through percolation networks, facilitated by sintering (Wei et al., 2022).

Naturally-derived support materials (e.g., silk, wax, and polysaccharides) typically succumb to enzymatic degradation, making resorption kinetics heavily dependent on implant location. Synthetic polymers are more susceptible to hydrolysis, the rate of which increases over time as water penetrates deeper into the material bulk, suddenly diminishing conductivity. Tuning of polymer crystallinity can slow the water diffusion. In some cases, oxidation aggravated by tissue inflammation can unpredictably reduce the operational lifetime of an implant (Feig et al., 2018).

Recently, the focus shifted towards exploring conjugated polymers as electroactive materials that are typically resistant to degradation via enzymatic cleavage or hydrolysis. They enable device integration deep into the tissue and undergo chain disintegration, leaving behind oligomeric fragments that could induce immune response (Tropp and Rivnay, 2021; Rai and Mantione, 2023).

Conventional implantable electronics includes electrodes such as rigid metallic wires or conductive structures on a stiff planar substrate. Typically, such electrodes are difficult to integrate with biological systems due to mechanical incompatibility, structural mismatch between soft tissue geometry and planar electrode surface, and poor attachment of non-flexible electrodes to moving or pulsating organs (Boufidis et al., 2025). These factors induce the foreign body response–tissue encapsulation of the implant–which changes near-electrode impedance, hindering sensing and stimulating capabilities.

**FIGURE 1:**
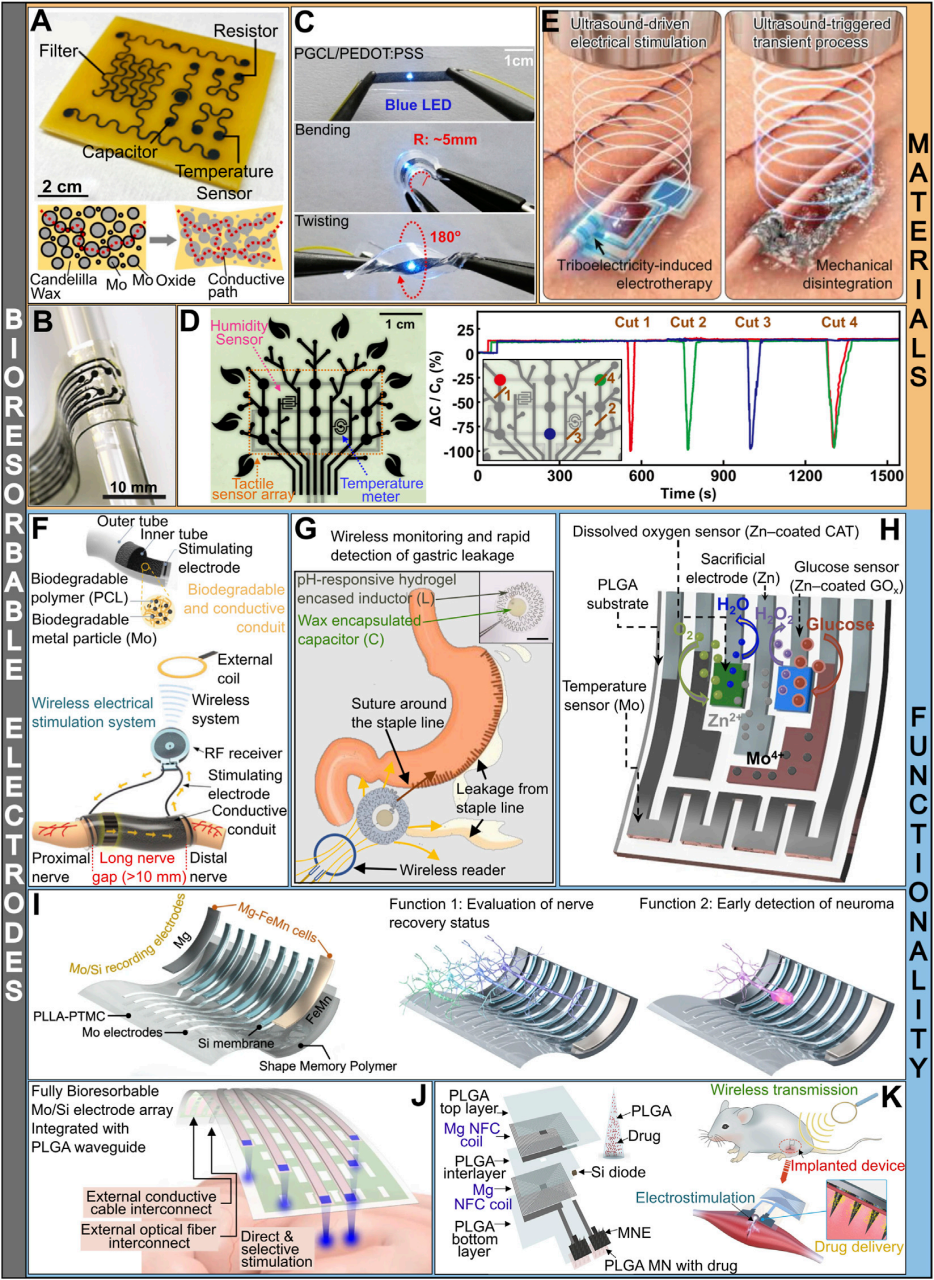
Bioresorbable electrodes: materials & functionality. **(A)** Multifunctional circuit with Candelilla Wax/Mo composite and schematic of the formation of conductive paths through self-sintering. Adapted with permission from (Wei et al., 2022), Copyright 2022, American Chemical Society. **(B)** Additive manufacturing of a flexible bioresorbable electrode array; substrate: poly (octamethylene maleate (anhydride) citrate) (POMaC), conductor: shellac-carbon ink. Adapted with permission from (Fumeaux and Briand, 2024), Copyright 2024, The Author(s). **(C)** Demonstration of electromechanical stability under external deformation modes of a conductive elastomeric composite PGCL/PEDOT:PSS. Adapted with permission from (Shin et al., 2024), Copyright 2024, The Author(s). LED: light-emitting diode. **(D)** Multimodal sensor array fabricated from self-healing elastomers as electrode substrate and conductive traces. Real-time capacitance measurement shows a fast autonomous restoration of electrical continuity after complete cuts at positions 1, 2, 3 and 4. Adapted with permission from (Jang et al., 2024), Copyright 2024, The Author(s). **(E)** Acoustically modulated operation and triggered degradation of a transient triboelectric nanogenerator for electrical nerve stimulation. Reproduced with permission from (D.-M. Lee et al., 2023), Copyright 2023, The Author(s). **(F)** Structure, materials and application of a biodegradable and conductive conduit for wireless nerve stimulation. Adapted with permission from (Kim Jio et al., 2023), Copyright 2023, The Author(s). **(G)** Schematic illustration of postoperative wireless monitoring of gastric leakage via the bioresorbable pH sensor and the device photograph (scale bar, 10 mm). Adapted with permission from (S. Li et al., 2024), Copyright 2024, The Author(s). **(H)** Fully printed, bioresorbable electrochemical sensor for continuous glucose monitoring with degradation compensation modules: a sacrificial electrode, temperature sensor and dissolved oxygen sensor. Adapted with permission from (Li et al., 2023), Copyright 2023, The Author(s). CAT: catalase. **(I)** Self-morphing neural interface for wireless interrogation of neuropathic injuries. Bioresorbable galvanic cell enables self-powered nerve growth stimulation, while the electrode array records signals for machine learning assisted evaluation of nerve recovery. Adapted with permission from (Wang et al., 2025), Copyright 2025, The Author(s). **(J)** Bioresorbable electrode array integrated with a polymer waveguide for simultaneous electrical recording and optical stimulation of the neural tissue. Adapted with permission from (Cho et al., 2024), Copyright 2024, The Author(s). **(K)** Schematic illustration of the structure and application of an implantable and bioresorbable microneedle device for simultaneous wireless electrostimulation and passive drug delivery. Adapted with permission from (Huang et al., 2022), Copyright 2022, American Chemical Society. NFC: near-field communication.

To address integration issues and eliminate surgical extraction, electrode components can be made from soft and degradable materials. Natural waxes and degradable elastomers, first introduced as electrode supports and protective coatings, are also suitable binders for bioresorbable metallic particles (e.g., Mo, W, Zn, Mg, and Fe). Candelilla wax (CW) was used as a hydrophobic matrix for direct loading of Mo microparticles, enabled by its low melting point (63 °C–65 °C) (Wei et al., 2022). The combined effect of matrix shrinkage and wax-facilitated corrosion of surface metal oxides, accelerable by immersion in phosphate-buffered saline (PBS) (pH 7.4, 37 °C), increased the conductivity to 1.4 × 10^4^ S/m. Under these conditions, electrical continuity was preserved for up to 19 days. Although this composite supported patterning of a resistive temperature sensor, a capacitive proximity sensor, and a low-pass filter (Figure 1A), wax brittleness limits the application in soft biological environments.

Conductive paste made from polybutylene adipate terephthalate (PBAT) and Mo microparticles achieved better electromechanical properties than CW/Mo composite. Adding tetraglycol (TG) as a lubricant to PBAT/Mo improved stretchability and increased the conductivity from 1,200 S/m to 1,800 S/m (Kim et al., 2022). Due to its excellent malleability, this paste can be used for printing and molding complex circuit components. Low-temperature fabrication and the elimination of manual assembly popularized additive manufacturing for transient electronics. Demonstrated printing platforms use a degradable elastomer poly (octamethylene maleate (anhydride) citrate) (POMaC) for transparent substrate/encapsulation and shellac-carbon ink for conductive traces (∼1,000 S/m) to fabricate electrode arrays with line widths of 200–300 μm and thicknesses of 30–50 μm (Figure 1B), potentially expediting the prototyping and optimization of transient electronic implants based on diagnostic imaging (Fumeaux and Briand, 2024).

Polymers used for surgical sutures and tissue engineering can be doped with conductive organic additives while retaining excellent elasticity. One example is a soft poly (glycolide-co-ε-caprolactone) (PGCL) matrix with a conductive poly (3,4-ethylenedioxythiophene)-poly (styrenesulfonate) (PEDOT:PSS) filler (Shin et al., 2024). Adding D-sorbitol induces the separation of conductive PEDOT-rich regions from PSS insulating chains, improving conductivity (∼6 × 10^4^ S/m). The composite retained its electromechanical integrity under different modes of mechanical load: bending (5 mm radius), stretching (100% strain), and twisting (180°) (Figure 1C).

Nevertheless, stretchability alone is insufficient to ensure reliable electrode performance under high mechanical stress. Dynamic reorganization of chemical bonds in polymers can promote fast recovery of conductive pathways after breaks and strong adhesion at the substrate-conductor interface (Figure 1D) (Jang et al., 2024). Substrate made of poly (lactide-co-ε-caprolactone)-diol (PLCL-diol), isophorone diisocyanate, and symmetrical disulfide derivatives as chain extenders exhibited self-healing behavior and strong adhesion to the PEDOT:PSS-based conductor. To achieve high conductivity (∼10^5^ S/m) and post-breakage recovery, PEDOT:PSS was doped with 1-butyl-1-methylpyrrolidinium bis(trifluoromethylsulfonyl) imide, poly (ethylene glycol) (PEG), and glycerol. Real-time capacitance measurements showed electrical continuity restoration within 1 min after complete cuts. Proof of concept for interrogation of moving organs was demonstrated on a mouse urinary bladder.

Adaptability to moving organs can be improved by using fiber electrodes, as their geometry allows for more secure attachment and better mechanical compliance. Electrode made of poly-ε-caprolactone (PCL) fiber core with surface-embedded Mo microparticles and an insulating layer (polypropylene carbonate or polyacrylic acid) showed good conductivity (2,252.6 S/m) and bending stability (against 4,000 cycles) (Kim Jinho et al., 2023). Melt-drawing of PCL fiber with subsequent cold-drawing to align polymer chains resulted in reduced flexural stiffness, while percolation network of Mo microparticles endowed conductivity. These structures can be applied as circuit interconnects for wireless temperature monitoring through RC coupling or for nerve stimulation. Fiber electrodes can also be incorporated into surgical procedures as functional sutures for short-term post-operative electrotherapy. Bioresorbable rope-like conductor made from twisted Mo wires coated with poly (lactic-co-glycolic acid) (PLGA) and PCL was used as a temporary epicardial pacing wire (TEPW) (Prieto et al., 2024). This structure demonstrated better mechanical compliance with the tissue than steel due to the Mo core, while the hydrophobic PCL outer layer delayed degradation, providing operation for at least 1 week. Electrical performance tests on Langendorff-perfused rat heart showed that degradable TEPW had comparable properties to conventional steel TEPW, regarding impedance, sensing amplitude, and slew rate of the QRS complex. Bioresorbable TEPWs are compatible with existing external pacemaker modules, facilitating the transition to real-life applications. However, long-term degradation effects on the impedance, signal quality, and stability of the TEPW were not investigated.

To avoid unpredictable signal interferences during passive electrode dissolution, externally controlled transiency of an otherwise stable encapsulation is favorable. Rapid, photo-triggered degradation of metastable cyclic poly (phthalaldehyde) (cPPA) coating was done by the conversion of near-infrared (NIR) light to heat by metal conductors (Istif et al., 2024). Sets of samples were exposed to 1 W of NIR light for different amounts of time (1–10 min) and for 3 min at different power levels (0.25, 0.5, 0.75, 1, and 2 W). Complete degradation of encapsulation was observed at 1 W/10 min and at 2 W/3 min. Required time for complete dissolution is determined by the composition of other electrode constituents. In case of the on-demand bioresorbable neurostimulator (Figure 1E), its operation and degradation were both acoustically modulated, using the power applied through commercially available devices (Lee et al., 2023). Poly (3-hydroxybutyrate-co-3-hydroxyvalerate) (PHBV) was used as support for a triboelectric composite PHBV/PEG:ChCl (ChCl-choline chloride). Neurostimulator could deliver stable, high-frequency (20 kHz) stimulation conditioned with 0.5 W/cm^2^ of acoustic power density spanning 5 days (5 min per day), while completely losing its function 20 min post-exposure to high energy waves (3 W/cm^2^). Importantly, it was validated that the initially produced disintegration fragments did not cause an immune response. However, migration of these particles could create obstructions, potentially causing serious complications.

## 3 Biomimetic electrode interfaces

Most bioresorbable devices rely on planar flexible and soft substrates to support the topology of the electrodes and achieve conformal interfacing with the tissues. However, the substrate limits access to deep tissue structures in targeted electrical stimulation and interfacing with electroactive tissues. An important step toward seamless 3D integration and amalgamation between electronics and biological tissues was achieved via the advances in ionic-electronic polymeric conductor fabrication. Injectable thiophene-based precursor formulations suitable for enzymatic and electrochemical polymerization *in vivo* enabled the formation of 3D biocompatible and stable or bioresorbable organic conductive gels with long-range conductivity (Strakosas et al., 2023; Hjort et al., 2023; Biesmans et al., 2024). These gels were successfully used to perform electrophysiological measurements and electrical stimulation of tissues, while the interfacing could be further localized to the level of individual cells using a two-step process involving initial monomer binding to the lipid membrane, followed by enzymatic copolymerization. Electrodes fabricated using the described strategies exhibited ohmic behavior and the ability to support continuous electric currents up to the ∼μA range or electrical stimulation pulses of ∼ms duration and relatively high amplitude (∼100 μA or ∼10 V), which was required to overcome electronic interfacing deficiencies. The design of 3D soft electrodes was mainly optimized for stability, biocompatibility, and tissue interfacing, while bioresorption engineering and assessment remain important topics for future studies.

## 4 Transient implants for electrotherapy

Electrostimulation therapy for traumatic nerve injury accelerates nerve regeneration and promotes functional recovery, especially if applied in the early post-operative period. This approach obviates risky autologous nerve grafting and uses multifunctional nerve conduits, which deliver structural guidance for axonal growth and electrical cues between damaged nerve ends. Rosenbalm et al. (2023) reported the use of high (10^–4^ S/m) and low conductivity (10^–6^ S/m) bioresorbable polymers, alternated in 600 μm segments, to produce repeated electric field gradients across the conduit, with or without external electrical conditioning. Yu et al. (2024) fabricated a multilayered nerve conduit, which had: 1) an inner layer made of aligned PCL fibers for axonal routing, 2) soft, middle layer made of poly (L-lactic acid) and poly (trimethylene carbonate), 3) randomly oriented PCL fibers as the outer layer, and 4) an integrated Zn-Mo battery which directly produced electrical cues for nerve growth. Electrophysiological studies were performed at 12 weeks postimplantation to assess the recovery of nerve conduction, which was comparable to the autograft control. Characterization of battery output was evaluated by constant current discharging in normal saline, with an output voltage around 0.5 V and discharge current density of 25 μA/cm^2^ for 50 h. Degradation effects *in vivo* and reproducibility of the stimulation were not evaluated.

Self-powered mechanism is particularly favorable for temporary electrotherapy, as it can provide stable, continuous current stimulation, with minimal effect on patient mobility and comfort. However, alternating current stimulation has more optimizable signal parameters and can be applied on demand during several days, which was demonstrated to be more effective compared to continuous stimulation. An inductive coupling-powered nerve conduit, made of a bioresorbable Mo/PCL composite, was used for treating sciatic nerve injury in a rat model (Figure 1F) (Kim Jio et al., 2023). Electrical pulses (100 μs duration, 20 Hz frequency) were administered for 1 h a day for 3 days following surgery, leading to significantly improved functional recovery. The optimization of stimulation parameters for nerve regeneration was not performed, and their effect on device stability and degradability was not thoroughly assessed. Further, non-degradable implanted radio frequency (RF) receiver should be substituted with a degradable module.

Apart from RF transmission, other external power transfer systems for electroceuticals include ultrasound triboelectric harvesters (Lee et al., 2023) and photovoltaics or phototransistors for simultaneous powering and control (Chen et al., 2023). A bioresorbable miniaturized pacemaker was also realized by combining Mg-MoO_3_ (or Zn-MoO_3_) battery electrodes as pacing electrodes with optical control (Zhang et al., 2025). NIR light was applied externally through the skin to a phototransistor to close the battery circuit, delivering electrical signals to the surrounding tissue. Output current was in the sub-mA range, with the triggering light intensity of up to 0.3 mW/mm^2^. Pacing thresholds have risen significantly from day 2 (Mg-MoO_3_) or day 19 (Zn-MoO_3_) due to local inflammation and electrode degradation. This system supports coupling with a wearable device that continuously monitors cardiac rhythm and optically triggers correction pulses when it detects arrhythmias (closed-loop operation) or integration with other implants for multi-site pacing modulated through wavelength-division multiplexing (e.g., for transcatheter aortic valve replacement). Given its small size and wire-independent functionality, the pacemaker could be adapted for other electrotherapy types and surgical procedures. This breakthrough technology shifts the perspective on temporary electroceuticals towards universally applicable miniaturized modules with on-demand wireless control, facilitating minimally invasive personalized therapy. However, to ensure effective and controlled treatment, detailed optimization and calibration of stimulation triggering is needed.

## 5 Bioresorbable physical and (electro)chemical sensors

Temporary and continuous localized monitoring during surgical procedures enables prompt corrective action in the case of otherwise indiscernible tissue microinjuries. Bioresorbable sensors can be designed to non-invasively record small variations in biomechanical signals by enhancing sensitivity via materials selection. Boutry et al. (2019) demonstrated a fully degradable arterial-pulse sensor based on fringe-field-capacitive sensing, making a significant innovation in wireless blood flow monitoring. Lee et al. (2024) reported a piezoresistive strain sensor for the recording of pulse pressure fluctuations on the blood vessel surface. Cracking of the Mo film resistor induced conductivity changes, achieving a high gauge factor of 1,355 at 1.5% strain. PCL substrate and MoO_3_ adhesion layer ensured crack-formation repeatability, while wax-based encapsulation enabled *in vivo* functionality for up to 3 days. From the fourth day, electrical conductivity rapidly diminished due to accelerated crack-induced layer dissolution.

In other cases, prolonged postoperative tracking is needed to detect asymptomatic post-surgical complications. Li et al. (2024) produced a gastric leakage detection sensor, consisting of a wax-encased polymer capacitor and a winding wire inductor embedded in a pH-responsive hydrogel (poly [2-(diisopropylamino)ethyl methacrylate] and poly (ethylene glycol)diacrylate copolymer), inductively coupled to an external readout (Figure 1G). Hydrogel swelling under acidic conditions induces expansion of the spiral inductor, changing the resonance frequency of the inductor-capacitor circuit. Response time is determined by hydrogen ion diffusion and water permeation to within 1 h, while the device showed stable *in vivo* operation up to 7 days, covering the relevant early-recovery period. *In vitro* assessment showed that following water penetration, metallic components dissolved faster, with wax and hydrogel gradually degrading over a longer timeframe (over 1 month).

Apart from post-surgical assessment, transient biosensors could be used as temporary diagnostic implants for precise organ activity mapping. Continuous interrogation of the cerebral cortex was done with multiplexed electrode arrays mounted on a shape-memory polymer substrate (PLCL-PLGA composite) (Bae et al., 2024). Web shape and substrate material enabled self-deployment into the intracranial space through a 5-mm syringe for a highly conformal contact. More accurate neurophysiological assessment implies monitoring of neurotransmitters alongside electrical signals. Electrochemical measurements are generally difficult to implement using degradable electrodes, due to unpredictable signal deterioration. However, Yang et al. (2022) demonstrated a transient platform for dopamine level recording alongside pH and temperature of deep brain regions. Dopamine oxidation was facilitated through electrostatic binding with atomically thin layers of MoS_2_ or WS_2_ functionalized with Fe nanoparticles. Sensing layers underwent gradual dissolution without adverse effects on cells, and the signal quality critically diminished after ∼2 weeks. Another approach to achieving signal stability are integrated compensation measurements. In a bioresorbable electrochemical glucose sensor (Li et al., 2023), additional electrodes measured the current drift due to temperature changes and material degradation, while a relatively stable dissolved oxygen concentration produced a current signal for real-time calibration (Figure 1H). Metal electrode arrays fabricated by screen-printing and low-temperature water sintering allowed continuous glucose sensing in the subcutaneous tissue (2 h per day for up to 5 days), showing a shorter operational cycle compared to commercial continuous glucose monitors. The current response of the oral glucose tolerance test in healthy rats attenuated by 34.91% and 72.76% on the third and fifth day, respectively. After 5 days, the signal-to-noise ratio decreased enough to show that compensation is insufficient for long-term glucose tracking without changes in material properties. To confirm if bioresorbable systems can appositely replace conventional non-degradable temporary sensing probes, rigorous, long-term investigation of effects of degradation products on signal integrity is necessary.

## 6 Bioresorbable multimodal electronic implants

Multifunctional electronic implants, combining sensing and therapeutic stimulation effects, enable simultaneous healing support and real-time recovery tracking. Wang et al. (2025) coupled a bioresorbable galvanic cell for electrotherapy and an electrode array for spatial tracking of nerve growth and early neuroma detection (Figure 1I). The device was implanted to treat long-gap nerve injury in rats, and data processing involved convolutional neural networks for advanced classification, correlating electrical signals with gait parameters or stumped nerve growth with sufficient accuracy. The galvanic cell and recording electrodes survived up to 7 weeks without major degradation. Electrical recordings remained reliable for up to 5 weeks, when signal integrity started to deteriorate due to electrode and galvanic cell dissolution.

In contrast to independent functionalities, localized stimulation applicable on-demand based on the sensor input from the same site enables adaptable and direct tissue manipulation. Cho et al. (2024) presented a platform for cerebral cortex interrogation, which combined electrophysiological recording with optogenetic stimulation (Figure 1J). A laser beam was delivered to the brain via a PLGA waveguide, while the Mo/Si bilayer electrodes were positioned underneath light exit points. Electrode grid design enabled light to pass to the targeted points, while the Mo overlayer eliminated photoelectric signal artefacts. Similar design and materials were employed in a transparent multichannel microelectrode array for cardiac electrical function mapping and pacing in combination with colocalized optical monitoring (Chen et al., 2023). While the possible interference effects between electrical and optical signals can be effectively minimized at the device design stage, degradation progression affects the rates of deterioration for key electrical and optical properties differently, thereby increasing the complexity of device reliability and failure analysis.

Multimodal therapy exploiting the joint effect of pharmaceuticals and electrostimulation could significantly expedite tissue recovery and reduce inflammation. Passive drug delivery is convenient to integrate with bioresorbable electrode structures, as demonstrated in Huang et al. (2022). Degradable microneedle electrodes (MNEs), made of PLGA sputter-coated with tungsten, were loaded with anti-inflammatory drugs and wirelessly conditioned to deliver electrical cues for muscle regeneration (Figure 1K). Further, triggered degradability enables better control of drug elution. Zhang et al. (2023b) demonstrated an array of drug reservoirs with a programmable opening mechanism. Photo-triggered activation of the phototransistor closed the battery circuit, allowing the anode valve to corrode and release the medication. Addressability of individual reservoirs was achieved via integrated optical filters, however, these, alongside phototransistors, were not resorbable. The number of delivery points discernible through filtering was limited by light scattering and absorption in biological tissues. Electrically modulated release could enable even more precise drug dosing to difficult-to-access locations in the tissue. A polymeric resistive heater was integrated into a multifunctional PGCL-based suture to induce controlled drug release via multiple cyclic pulses (Shin et al., 2024). Wireless heating actuation for on-demand drug delivery was used in a multifunctional mesh for wireless pressure monitoring of a hernia-repair site. The synergistic effect of prolonged mechanical support and targeted administration of antibacterial medication could reduce the risk of hernia recurrence (Kaveti et al., 2024). Multimodal systems exploiting different strategies for direct or indirect electrical modulation of drug delivery can enable the triggering of passive release, on-demand drug elution, and even some degree of real-time control over operational lifetimes from days to months. Activation of drug release typically accelerates device degradation, and often leads to irreversible changes in material properties inducing higher drug delivery rates. These systems would benefit from innovative mechanisms for targeted and reversible triggering of degradation-mediated drug release and strategies for preventing passive drug elution in critical applications.

## 7 Summary, challenges, and perspectives

Bioresorbable electrodes are steadily developing, driven by the progress in materials science and facile fabrication techniques. Although the library of available resorbable materials remains limited, reliable electrodes exhibiting advanced features (e.g., self-healing behavior, triggered transiency, controllable longevity, and *in vivo* self-assembly) were successfully fabricated in various geometries, such as fibers and meshes, flexible planar arrangements, and 3D soft networks interfacing deeper tissue structures. They found uses in diverse short-term non-chronic applications (typical life cycle of several days), including electrical stimulation, electrophysiological monitoring, physical and (bio)chemical sensing, drug delivery, and even multifunctional devices. Electrode operation was effectively powered using galvanic cell-based approaches or wireless energy transfer via inductive or optical coupling. Similar coupling strategies were utilized to trigger targeted and addressable electrode activity or induce electrode-mediated processes. Notable methods to compensate for electrode degradation were also introduced.

Regardless of significant advances in bioresorbable electrodes, multiple key challenges remain to be addressed. Seamless and targeted 3D integration with soft living tissues allowing power delivery and access to deeper tissue structures, is still in its infancy, and the long-term tissue response to such systems remains unexplored. Despite the emerging capabilities of on-demand transiency, degradation processes and their concomitant effects (e.g., fragmentation and toxicity) still need to be thoroughly investigated to achieve sufficient control throughout the electrode life cycle. Future studies should also prioritize a more detailed assessment of the interplay between degradation evolution and electrode functionality, which is crucial for validation of device safety and reliability. Reliable electrode operation time frame, often limited to a period of up to 2 weeks, needs to be extended to up to a few months to address complex continuous monitoring and stimulation applications. In this case, protocols for sensor calibration and optimization of actuation parameters are needed to ensure stable and reproducible operation. Finally, to advance the theranostic use toward highly multiplexed systems, innovative design, fabrication, and surface modification approaches are required for electronic, multisensory, and multimodal integration of electrode arrays. Concurrent operation of different modules in multipurpose bioresorbable implants could have a synergistic effect on degradation kinetics, which needs to be studied in more detail.

Bioresorbable electrodes are continuously evolving and aiming to reach the levels of functionality comparable to or beyond their non-degradable counterparts. The progress in *in vivo* polymerization, electrode fabrication, functionalization strategies, and bioresorbable power supplies will continue to drive the field forward through the focused efforts of scientific, engineering, and clinical communities.
